# Profile of patients with hepatocellular carcinoma: An experience from a tertiary care center in India

**DOI:** 10.1007/s12664-021-01209-0

**Published:** 2022-02-28

**Authors:** Balaji Musunuri, Shiran Shetty, Ganesh Bhat, Karthik Udupa, Ananth Pai

**Affiliations:** 1 Department of Gastroenterology and Hepatology, Kasturba Medical College, Manipal, Manipal Academy of Higher Education, Manipal 576 104, India; 2 Department of Medical Oncology, Kasturba Medical College, Manipal, Manipal Academy of Higher Education, Manipal 576 104, India

**Keywords:** BCLC grading, Etiological profile, Hepatitis B, Histocytopathology profile, Liver cancer surveillance, NAFLD

## Abstract

**Background:**

The prevalence of hepatocellular carcinoma (HCC) is increasing worldwide and it is now the third most common cause of cancer-related death. HCC is becoming a major health burden with steadily increasing incidence globally.

**Methods:**

This is an observational study over a 3-year period in a tertiary care center in India. Three hundred and thirty-nine patients diagnosed to have HCC were included in this study. Patients’ clinical, etiological, radiological and cytohistological data and therapy offered were recorded and analyzed.

**Results:**

Cirrhosis of the liver was seen in 73.2% of the patients. 16.8% of patients were asymptomatic at the time of presentation. Ascites (57.2%) and jaundice (22.4%) were the most common signs of hepatic decompensation. The most common etiology of HCC was cryptogenic/non-alcoholic fatty liver disease (NAFLD) in 51% of the patients, while hepatitis B and C were seen in 17.4% and 5.8% of the patients, respectively. Advanced and end-stage disease with Barcelona Clinic Liver Cancer (BCLC) stages C and D were seen in 62.4% of patients. 56.6% had Albumin-bilirubin (ALBI) score of 2, while 62.8% had Okuda stage II disease. High alpha-fetoprotein (AFP) levels (>400 ng/mL) were seen in 48.9% of patients. Macrovascular invasion and metastases were seen in 45.9% and 22.2% of the patients, respectively. 17.6% of patients had evidence of tumor thrombus. 14.5% of biopsy specimens showed associated steatosis/steatohepatitis along with confirmation of HCC. Only 26.6% of the cirrhotic HCC patients were diagnosed during surveillance.

**Conclusions:**

HCC due to unknown cause/NAFLD appears to be overtaking hepatitis B as the commonest cause for HCC. Despite the advances in diagnostic methods and surveillance, most cases of HCC tend to be diagnosed at advanced stages.

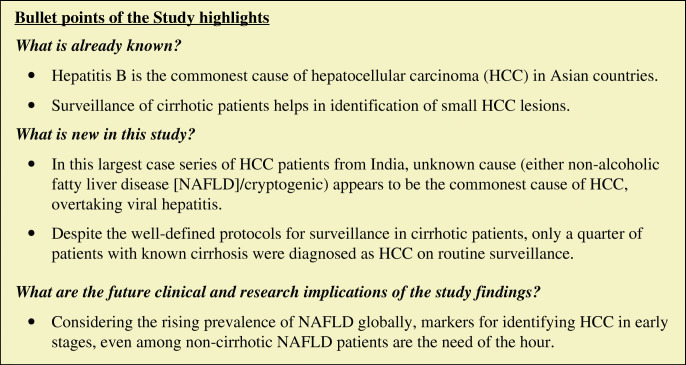



## Introduction

The incidence and prevalence of hepatocellular carcinoma (HCC) is rising, mainly due to the epidemic of non-alcoholic fatty liver disease (NAFLD), and is poised to become the leading cause of liver cancer [1]. The 2018 statistics from Global Cancer Observatory of World Health Organization (WHO) estimated that liver cancer was the sixth most commonly diagnosed cancer worldwide, with fourth leading cause of mortality among cancers [2]. Data on the epidemiology of HCC from India are sparse, and of variable and uncertain quality [3, 4]. The incidence of HCC in cirrhotics in India was observed to be 1.6% per year [5]. With this background, we conducted this retrospective study to know the clinical, etiological, radiological, and histological profile of patients with HCC.

## Methods

This is a retrospective cross-sectional study of prospectively maintained data, conducted at a tertiary care center in India. Institutional Ethical Committee (IEC)   clearance was taken for the study (IEC: 410/2020). The clinical records of all patients admitted with  HCC at our center between March 2017 and March 2020 were reviewed. All the patients who fulfilled  the diagnostic criteria of  HCC according to European Association for the Study of the Liver (EASL) guidelines were taken into study [6]. Data were collected in a predesigned questionnaire. Patients with incomplete and missing data were excluded from the study.

All patients’ details of clinical presentation, history of alcohol consumption, presence of cirrhosis, duration of cirrhosis (if known cirrhotic), blood investigations including complete blood counts, liver function tests, hepatitis B surface antigen (HBsAg) by chemiluminescence method, anti-hepatitis C virus (HCV) antibody by chemiluminescence method, alpha-fetoprotein (AFP) by enzyme chemiluminescence immunoassay (ECLIA) method, radiological features including tumor size, number, and location were recorded. Details of biopsy specimen and therapy offered were also recorded. Diagnosis of cirrhosis was made on the basis of clinical, biochemical, endoscopic, and radiological findings. Hepatitis B virus (HBV)-related cirrhosis was diagnosed when HBsAg was detectable in serum. HCV-related cirrhosis was diagnosed when detectable anti-HCV, HCV ribonucleic acid (RNA) (reverse transcription polymerase chain reaction [RT-PCR] by COBAS TaqMan HCV Test v2.0 or Cepheid Xpert HCV viral load kit), or both were present in serum.

Etiology of cirrhosis was considered to be alcohol, if the alcohol consumption was more than 40–80 g/day for males and 20–40 g/day for females for more than10 years [7]. Severity of cirrhosis was graded based on the Child-Turcotte-Pugh (CTP) classification and model for end-stage liver disease (MELD) score [8]. All the patients with cirrhosis at our center were on regular surveillance for HCC as per standard protocol as per EASL guidelines.

Diagnosis of HCC was based on non-invasive criteria with typical findings of HCC in triple phase contrast multidetector computed tomography (MDCT)/magnetic resonance imaging (MRI) and/or histopathology. Image characteristics of liver lesion of arterial phase hyperenhancement according to LI-RADS (Liver Imaging Reporting and Data System) classification and washout on portal venous and/or delayed phases were diagnosed as HCC [9]. Complete or partial non-opacification of part of, or whole, portal vein and its branches during portal venous phase was considered as thrombus. Similarly, enhancement of the walls of the portal vein in the presence of thrombus was considered as tumor thrombus. Imaging findings were interpreted by two radiologists. Tumor characteristics of size, site and number of lesions, presence of tumor thrombosis, and metastases were recorded. In case of any disconcordant radiological findings, patients underwent biopsy from liver lesion for characterization. Ultrasound-guided percutaneous biopsy of the liver lesion was done by a radiologist wherever necessary and processed with standard histological staining techniques. Immunohistochemical staining with arginase-1, HepPar-1, cytokeratin-7 (CK7), CK20, etc. were used wherever necessary to confirm HCC and distinguished from secondaries and non-HCC tumors. Staging was done according to the Barcelona Clinic Liver Cancer (BCLC) staging classification, Okuda staging, and Albumin-Bilirubin (ALBI) grading [10–12]. Patients were discussed in multi-disciplinary tumor board and offered therapy as per performance status and BCLC staging.

Categorical variables were presented as percentages, while continuous variables were presented as mean (standard deviation) or as median (interquartile range). Comparisons between proportions were performed using the Chi-square test and continuous variables using the student *t* test and Mann-Whitney non-parametric *U* test. For all tests, *p values* < 0.05 were considered statistically significant. The analysis was performed using Statistical Package for the Social Sciences (SPSS) software (IBM SPSS Statistics for Windows, Version 26.0. IBM Corp., Armonk, NY, USA).

## Results

During the study period of 3 years, a total of 3038 patients with diagnosis of chronic liver disease (CLD) were admitted. Three hundred and forty-nine patients of HCC with 541 admission events were noted. A total of 339 patients with HCC  were included.

### Clinical characteristics

**Fig. 1:**
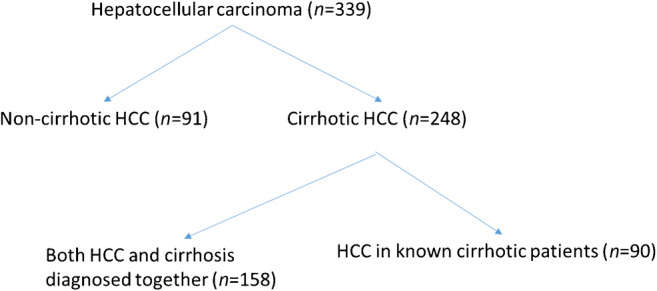
Nature of hepatocellar carcinoma (HCC) patients in  respect to underlying cirrhosis

The mean age was 62.8 ± 10.2 years, 66.3% of the patients were above 60 years. Majority (91.1%) of the patients were male. 73.2% (248/339) of the patients with HCC had a background of cirrhosis on imaging (ultrasonography [USG]/computed tomography [CT]/MRI) at the time of diagnosis, while the remaining 26.8% (*n*=91) of HCC patients were non-cirrhotic. One hundred and fifty-eight patients (63.7% of cirrhotic HCC ) were detected to have HCC and cirrhosis on the first presentation. In the remaining 90 patients, HCC was detected during the follow-up, either due to surveillance (24 patients, 9.6% of all cirrhotic HCC) or due to symptoms during follow-up (66 patients, 26.6% of all cirrhotic HCC). Among the surveillance group, the median duration from diagnosis of CLD to detection of HCC was 35.5 months (Fig. 1).

16.8% of HCC patients (*n*=55) were asymptomatic or were diagnosed incidentally, when being evaluated for unrelated illness, of whom 40 patients were cirrhotic, while the remaining were non-cirrhotic. The most common clinical presentations were abdominal pain (24.4%), ascites (24.1%), and anorexia (20%) followed by weight loss (17.9%) and fatigue (10.9%) (Table 1).

**Table 1:** Clinical characteristics of patients with hepatocellular carcinoma

Clinical parameters	number (%)
Age (y)	62.8 (10.2)
Male	309 (91.1%)
Non- cirrhotic HCC	91 (26.8%)
First presentation as HCC	249 (73.4%)
**Comorbidities**: DM/HTN (%)	44.2 / 32.4
**Clinical manifestations:**	
Asymptomatic	16.81
Abdominal pain	24.4
Ascites	24.1
Anorexia	20
Weight loss	17.9
Fatigue	10.9
GI bleed	6.4
Jaundice	6.1
Abdominal mass	1.7
Other symptoms (bony pain, hemoptysis, DVT, fever, diarrhea)	2.9
**Clinical signs:**	
Pallor	13.8
Icterus	22.4
Ascites	57.2
Moderate-gross ascites	35.6
Hepatic encephalopathy	0.9

Continuous variables were expressed as mean (S.D.); categorical variables were expressed as *n* (%). *SD* standard deviation, *DM* diabetes mellitus, *HTN* hypertension, *DVT* deep vein thrombosis, *GI* gastrointestinal; clinical manifestations and clinical signs were expressed in %,* HCC* hepatocellar carcinoma

Signs of hepatic decompensation including ascites, jaundice, gastrointestinal (GI) bleed, and hepatic encephalopathy were noted in 194 (57.2%), 76 (22.4%), 22 (6.4%), and 3 (0.9%) of patients, respectively. Eleven patients (3.2%) had spontaneous rupture of HCC at first presentation.

### Etiology and co-morbidities

The most common cause of HCC was unknown/cryptogenic in 51% of the patients, while HBV and HCV constituted 17.4% and 5.8 % of the causes, respectively. Significant alcohol intake was seen in 19.4% of the patients. Rest of the etiology were constituted by combination of factors: alcohol + HBV, alcohol + HCV and HBV + HCV in 3.2%, 1.1% and 0.5% of patients, respectively (Table 2).

**Table 2:** Etiological profile of hepatocellular carcinoma

Etiology	%
Unknown	51.3
Alcohol	19.4
Hepatitis B	17.4
Hepatitis C	5.8
Alcohol and hepatitis B	3.2
Alcohol and hepatitis C	1.1
Hepatitis B + hepatitis C	0.5
Others (Wilson disease, AIH, GSD)	0.8

*AIH* autoimmune hepatitis, *GSD* glycogen storage disorder

Among non-cirrhotic HCC patients, 82.4% (75/91) had no obvious precipitating factor, while HBV accounted for 8.9% and significant alcohol was seen in 7.6%.

Among cirrhotic HCC patients, the most common cause was unknown in 39.9%. Significant alcohol was seen in 23.7%, while viral etiology of HBV and HCVwas seen in 20.5% and 7.6% of patients. A combination of factors (alcohol + HBV, alcohol + HCV and HBV + HCV) was seen in 6.85% of cirrhotic patients.

Diabetes mellitus (DM) was present in 44.2% of the patients, while hypertension (HTN) was seen in 32.4%, ischemic heart disease in 8.5%, hypothyroidism in 2.9%, cerebrovascular accident (CVA) in 3.8%, chronic kidney disease (CKD) in 3.2%, and chronic obstructive pulmonary disease (COPD) in 3.5%. Among the cryptogenic group, 51.1% and 41.3% of the patients had DM and HTN, respectively. Three patients had human immunodeficiency virus (HIV) infection, two among the HBV group and one among the unknown group.

### Blood parameters

Normal AFP levels up to 20 ng/mL were seen in 27.4% of patients, while AFP levels more than 400 ng/mL were seen in 48.9% of patients. Coagulopathy with international normalized ratio (INR) > 1.5 was seen in 14.5% of patients. Elevated aspartate aminotransferase (AST) and alanine aminotransferase (ALT) levels more than twice the upper normal limit were seen in 45.1% and 12.7% of patients. Elevated ALP levels more than two times the upper normal limit were seen in 49.3% of patients (Table 3).

**Table 3:** Blood parameters in patients with hepatocellular carcinoma

Parameter	
Hb (g/dL)	11.3 (2.1)
TLC (cells per mm^3^)	7300 (2900–26400)
PLT (cells per mm^3^)	1.645 (0.2500–17.1)
TB (mg/dL)	1.500 (0.17–24.10)
Alb (g/dL)	3.280 (1.7–4.9)
AST (U/L)	77.00 (4–810)
ALT (U/L)	39 (6–731)
ALP (U/L)	157.0 (40–1187)
AST (> 2 times UNL) (%)	45.1%
ALT (> 2 times UNL) (%)	12.7%
ALP (> 2 times UNL) (%)	49.3%
INR >1.5 (%)	14.5%
AFP (ng/mL)	353.3 (0.8–395444)
AFP < 20 ng/mL (%)	27.4%
20–400 ng/mL (%)	23.7%
400–1000 ng/mL (%)	8.3%
>1000 ng/mL (%)	40.6%

*Hb* hemoglobin expressed in g/dL mean (SD), *TLC* total leukocyte count, *PLT* platelet count, *TB* total bilirubin, *Alb* albumin, *AST* aspartate aminotransferase, *ALT* alanine aminotransferase, *ALP* alkaline phosphatase, *INR* international normalized ratio, *AFP* alpha- fetoproteinin, *UNL* upper normal limit, TLC PLT, TB, Alb, AST, ALT, ALP and AFP expressed as median (interquartile range), % of patients with AFP levels <20, 20–400, 400–1000, and >1000 ng/mL

HBV viral load was available for 32 patients, among whom 53.1% of patients had levels more than 10^5^ IU/mL. Majority of patients had hepatitis B e antigen (HBeAg) negative (24 out of 26 patients).

### Tumor characteristics

Most of the patients were having Child-Pugh category B followed by A in 45.4% and 41%, respectively. Majority belonged to BCLC C (48.8%) followed by B (25.4%). Very-early/early-stage disease (BCLC-0 and A) was seen in 12.1% of patients, and advanced stage/end-stage disease (BCLC-C and D) was seen in 62.4% of all patients. 56.6% had ALBI score of 2, while 62.8% had Okuda stage II disease (Table 4).

Right lobe involvement was seen in 51.2% and bilobar involvement in 32.3% of the patients. 55.5% of the patients had multiple lesions, while 60.5% of the patients had lesion more than 5 cm at the time of presentation. The mean size of the lesions was 7.5 ± 4.6 cm. Macrovascular thrombosis was seen in 45.9% of the patients, while 22.2% of the patients had metastatic disease at presentation. 67.5% of the thrombosis was limited to portal vein, 25.9% had thrombosis involving infradiaphragmatic vessels apart from portal vein (splenic vein, superior mesenteric vein, hepatic veins, and infradiaphragmatic inferior vena cava [IVC]), while the rest 6.4% had involvement of supradiaphragmatic IVC and/or extension to the right atrium. 17.6% (60 patients) of the patients had radiological evidence of tumor thrombosis.

The most common site of metastases were lungs in 62.6%, followed by liver and bone in 16% and 13.3%, respectively. Other organs included peritoneum (6.6%), adrenal gland (6.6%), brain (4%), spleen (2.6%), and pancreas/kidney (1.3% each) (Table 4).

**Table 4:** Tumor characteristics and severity of disease

Parameter	
CTP: A/B/C	41.0/45.4/13.6
MELD	11 (9–15)
BCLC: 0/A/B/C/D	1.7/10.3/25.4/48.8/13.6
ALBI: 1/2/3	14.7/56.6/28.6
Okuda: I/II/III	17.7/62.8/19.5
Location of tumor	
Right lobe/left lobe/bilobar	51.2/16.3/32.3
Number of lesions	
Single/two/multiple	33.4/11.4/55
Size of the lesion	
< 2 cm/2–5 cm/> 5 cm	5.7/33.7/60.6
Size of lesion (in cm)	7.594 (4.628)
Macrovascular thrombosis	155 (45.7%)
PV thrombosis	67.5%
Infradiaphramatic thrombosis	25.9%
Supradiaphragmatic thrombosis	6.4%
Tumor thrombosis	60 (17.6%)
Metastasis	75 (22.1%)

*CTP* Child-Turcotte-Pugh score stages A, B, and C expressed as percentages, *MELD* model for end-stage liver disease score expressed as median (IQR), *BCLC* Barcelona Clinic for liver cancer staging, *ALBI* albumin bilirubin grading. BCLC stages, ALBI stage, and Okuda stage were expressed as % of overall patients. Size of the lesion was expressed as mean (SD); rest of the tumor characteristics were expressed as percentages of overall patients

### Therapy offered

Treatment details were available for 264 patients. Therapy with curative intent was offered to 13.6% of patients. Surgical resection was offered to 16 (6%) patients, of whom 4 underwent transarterial chemoembolization (TACE) prior to resection. Radiofrequency ablation was offered to 19 patients (7.1%). TACE was offered to 13.2% (*n*=35) of patients. One patient underwent transarterial radioembolization (TARE) and another patient underwent liver transplantation. Palliative stereotactic body radiotherapy (SBRT) was offered to 3 patients and one patient received palliative radiotherapy to vertebral metastasis for symptom relief. Systemic chemotherapy with sorafenib was offered to 54.1% (*n*=143) of patients. Palliative therapy was given to 21.9% (*n*=58) of patients.

### Histopathology profile

USG-guided tissue acquisition from liver lesion for histopathological confirmation was done in 69 patients, of whom 5 underwent fine needle aspiration (FNA). Four patients among the FNA group had findings suggestive of HCC, while one with inadequate sample required repeat biopsy for characterization of the lesion. Sixty-one patients underwent USG-guided percutaneous liver biopsy, while one underwent intraoperative liver biopsy and two underwent biopsy from skeletal metastatic lesions. Thirty-five biopsy specimen were processed with additional immunohistochemistry markers like arginase-1, HepPar-1, and others like CK7 and CK20. All biopsy results except for one patient (who underwent repeat biopsy) yielded positive report. Among these biopsy specimen, 7 had evidence of macrosteatosis and 3 had steatohepatitis, while four patients showed evidence of liver fibrosis along with findings of HCC.

### Comparison of cirrhotic vs non-cirrhotic HCC

On comparing various parameters among cirrhotic and non-cirrhotic HCC patients, it was found that there were statistically significant differences across various stages of HCC including BCLC staging (*p*=0.0001), ALBI (*p*<0.001), Okuda scoring (*p*=0.036), and CTP class (*p*<0.001). Platelet-to-lymphocyte ratio (PLR) was found to be significantly lower in the group of cirrhotic HCC. AFP levels were comparable in both the groups. Patients in non-cirrhotic HCC group were having higher age, larger size of the lesion, and lower MELD score compared to the cirrhotic HCC group (Table 5).

**Table 5:** Differences between cirrhotic and non-cirrhotic hepatocellular carcinoma patients

Parameter	Cirrhotic HCC	Non-cirrhotic HCC	*p* value
Age (years)	61.78 ± 9.51	65.57 ± 11.52	0.002%
Size (cm)	6.22 ± 3.78	11.32 ± 4.67	< 0.005%
BCLC–O/ A/ B/ C/ D	6/ 29/ 50/ 121/ 42	0/ 6/ 36/ 45/ 4	< 0.005*
ALBI–1/ 2/ 3	20/ 139/ 89	30/ 53/ 8	< 0.005*
Okuda–I/ II/ III	40/ 152/ 56	20/ 61/ 10	0.036*
CTP–A/ B/ C	77/ 129/ 42	62/ 25/ 4	< 0.005*
MELD	13.22 ± 5.22	10.23 ± 4.04	< 0.005*
AFP (ng/mL)	13877 ± 39190	17401 ± 35836	ns %
NLR	4.25 ± 3.38	5.02 ± 5.09	ns %
PLR	132.11 ± 94.92	230.72 ± 168.55	< 0.001%
Thrombosis–yes/ no	121/ 125	61/ 30	0.007*

Variables expressed as % of total patients according to stages of BCLC, ALBI, Okuda, and CTP. AFP levels (in ng/mL), NLR (neutrophil-lymphocyte ratio), and PLR (platelet lymphocyte ratio) were expressed as mean ± SD

*Chi-square test, % independent student t-test; *p*<0.05 is significant; ns=not significant

*BCLC* Barcelona Clinic Liver Cancer staging, *ALBI* Albumin-Bilirubin grading, *CTP* Child-Turcotte-Pugh score, *MELD* model for end-stage liver disease score, *AFP* alpha- fetoprotein, *NLR* neutrophil-lymphocyte ratio, *PLR* platelet lymphocyte ratio

### Correlation of AFP levels, NLR and PLR

AFP levels were found to correlate significantly with size of lesion (*r*=0.1239, *p*=0.0411), presence of thrombosis (*p*<0.0001), and across BCLC stages (*p*<0.0001) and Okuda stages (*p*=0.03069).

There was statistically significant correlation of neutrophil-to-lymphocyte ratio (NLR) with presence of metastases (*p*=0.026), size of lesion (r= 0.1945, *p*= 0.0016), MELD (r= 0.2572, *p*= 0.0001), CTP score (r= 0.2182, *p* < 0.0001), BCLC stages (*p*=0.0002), Okuda (*p*<0.0001), and ALBI staging (*p*=0.005). However, it did not correlate with AFP levels (*r*= 0.04529, *p*= 0.4291) and with respect to presence of thrombus (*p*= 0.1356). Similarly, PLR was found to be significantly different with presence of metastases (*p*=0.001), size of lesion (*r*= 0.4874, *p* < 0.0001), BCLC, and ALBI staging. However, there was no correlation of PLR with respect to AFP (*p*= 0.2189), CTP (*p*= 0.2809), and MELD score (*p*= 0.3141).

## Discussion

Liver cancer is predicted to be the sixth most commonly diagnosed cancer and the fourth leading cause of cancer death worldwide in 2018 [2]. HCC is the most common primary malignant liver tumor accounting for approximately 75% to 85% of the primary hepatic malignancies [2]. Almost a third of those with cirrhosis will develop HCC during their lifetime [6, 13].

In our study, 61.6% of patients belonged to age group of 60–80 years and 32.1% belonged to the age group of 40–60 years. Male-to-female ratio was 10.3:1. The age-specific incidence is different in different parts of the world [14, 16]. The incidence of HCC is higher in men and in those over 40 years old [17]. The incidence of HCC increases progressively with advancing age in all populations, reaching a peak at 70 years [18].

Non-cirrhotic HCC accounts for 26.8% of all patients in our study, while the others were having a background of cirrhosis. In various studies done worldwide including India, cirrhosis was seen in 60% to 99% of patients with HCC [14, 15, 19, 20, 22].

Abdominal discomfort/pain, abdominal distention, and anorexia were common presenting complaints among our patients. This is similar to other studies from India, where abdominal pain and distension were predominant presenting symptoms [14, 15, 23].

The etiological factors for HCC vary in different geographical regions. No cause of HCC was evident in 51.3% of all patients. No obvious cause was found in 82.4% of non-cirrhotic HCC patients and 39.9% of cirrhotic-HCC patients. Most of the patients among the group of unknown etiology had associated DM (51.1%) and HTN (41.3%). The high prevalence of DM and HTN among patients with unknown probably points towards NAFLD as an etiology. The lack of biopsy from  non-tumorous liver parenchyma for all patients was a barrier in identifying the true prevalence of NAFLD among our patients. Our study being retrospective in nature, the risk factors for NAFLD could not be assessed. NAFLD-associated HCC is more likely to arise even in the absence of cirrhosis. It is estimated that half of the cases of non-alcoholic steatohepatitis (NASH)-induced HCC arise in non-cirrhotic patients [26–29].

Viral infection with HBV and HCV was seen in 17.4% and 5.8% of all HCC patients. Viral hepatitis was seen in 9.8% of non-cirrhotic patients, while it was seen in 28.2% of cirrhotic HCC patients. Significant alcohol intake was seen in 19.4% of all patients and accounting for 7.6% of non-cirrhotic HCC and 23.7% of cirrhotic HCC patients. Combination of factors were seen in 3.2% (alcohol + HBV), 1% (alcohol +HCV), and 0.5% (HBV + HCV) of all patients, and all were having cirrhosis.

Our study findings were in contrast to the findings from other reported series from India, which showed viral hepatitis (HBV and HCV) to be commonest etiology for HCC [14, 15, 21, 23–25]. Chronic alcohol use of more than 80 g per day for longer than 10 years increases the risk for HCC by fivefold [26]. Moreover, huge regional differences in the prevalence of HBV and HCV infection might exist in India (i.e. the prevalence of HCV infection is highest in the Punjab). In another study from southern India, 85% of the patients with non-B non-C HCC had at least one risk factor for NAFLD [33]. These differences might translate into large differences in the incidence of HCC between states. Ten out of 69 histological examination of biopsy revealed associated steatosis and steatohepatitis. The lack of biopsy of background liver along with liver lesion precludes exact diagnosis of NAFLD among such patients.

Macrovascular invasion and metastases were seen in 45.7% and 22.1% of patients, while tumor thrombus was noted in 17.6% of patients. AFP > 400 ng/mL was seen in 48.9% of patients. Only 12.4% of patients had very early and early stage disease (BCLC- 0 and A) amenable for curative treatment. The nature of lesions including macrovascular invasion and metastases was similar to other studies from India [14, 15, 23–25]. 17.6% of our patients had evidence of tumor thrombus at presentation. Portal vein tumor thrombosis (PVTT) is known to occur in about 10% to 40% of patients at first diagnosis in various studies [27].

Our study shows that both NLR and PLR correlate significantly with the presence of metastases and size of lesion and with BCLC and ALBI staging, however not correlating with AFP levels. AFP levels were found to correlate with size of the lesion, presence of thrombosis, and across BCLC and Okuda stages. Both the NLR and PLR were identified as predictors of overall survival and recurrence-free survival [28].

Only 26.6% (24/90) of known cirrhotic HCC patients were diagnosed during surveillance and had median period of 35.5 months of cirrhosis prior to detection of liver lesion. In an American study, only 22% of cases known to have cirrhosis had undergone HCC screening prior to diagnosis [29]. In a study to understand failure rates in surveillance, the surveillance was reported to be more likely among patients seen by hepatologists and less likely in those with alcohol abuse [30].

Spontaneous rupture of HCC was seen in 3.2% of patients in our study. To our knowledge, this is the first study which noted prevalence of rupture of HCC among Indian patients as there are only few case reports and series. In recent reports from Italy and China, the reported incidence of rupture of HCC varies from 3% to 4.8% [31, 32].

In conclusion, the majority of patients with HCC present at an advanced-stage limiting the therapeutic options that can be offered. NAFLD probably is becoming the most common etiological factor among both cirrhotic and non-cirrhotic patients of HCC. These findings must be interpreted in light of the limitations of the present study being a cross-sectional study with no follow-up. Current surveillance protocol and its compliance need to be assessed in a prospective study to validate the benefit of surveillance in these patients.

## Declarations

### Disclaimer

The authors are solely responsible for the data and the contents of the paper. In no way, the Honorary Editor-in-Chief, Editorial Board Members, the Indian Society of Gastroenterology or the printer/publishers are responsible for the results/findings and content of this article.
